# English Review

**Published:** 1891-11

**Authors:** 


### *ENGLISH.*

### A Case of Superfœtation in a Mare.

Prof. Malet.

[*The Journal of Comparative Pathology and Therapeutics.*]

In the *Revue Vetérinaire* for August, 1891, Professor Malet records a very interesting case, which proves incontestably the possibility of superfœtation in the mare. As a rule impregnation puts a stop to œstrum, and that does not re-appear until after parturition. According to M. Saint-Cyr, the female domestic animals refuse the male six to eight days after a fruitful copulation, but, as Professor Malet remarks, exceptions to this rule are not rare, and well known to breeders. Mares that have already conceived frequently take the stallion readily, even although the pregnancy has already lasted for several months, and the same is seen in the cow, but more rarely. The question whether impregnation may follow such a second copulation has been disputed. It is necessary to draw a distinction between this phenomenon, which is properly termed *superfœtation*, and two others with which it is sometimes confounded, viz., *simultaneous fecundation*, and *superfecundation*. Simultaneous fecundation is applied to cases in which several ova simultaneously extruded are fertilised by the same male, while superfecundation denotes the fertilisation of ova of the same period by different males.

The following case is undoubtedly an example of superfœtation :

A mare, aged 14 years, was covered with a donkey on the 18th, 19th, and 25th of March, and the 8th of April, 1890; and then with a stallion on the 8th of May. These dates were registered in the establishment to which the mare belonged, and they have been verified by Professor Malet. Eleven months after the first service by the donkey, and ten months after the single service by the stallion, the mare gave birth to two young, viz., a mule and a foal. Parturition was laborious, owing to a mal-presentation of the mule. This mule, which was small for the breed, was never able to stand alone, but it lived for three days. The foal, born dead, was expelled without difficulty. Its development was incomplete, the only hairs present being on the tail and mane.

